# Tetramethoxychalcone, a Chalcone Derivative, Suppresses Proliferation, Blocks Cell Cycle Progression, and Induces Apoptosis of Human Ovarian Cancer Cells

**DOI:** 10.1371/journal.pone.0106206

**Published:** 2014-09-02

**Authors:** Zihao Qi, Mingming Liu, Yang Liu, Meiqin Zhang, Gong Yang

**Affiliations:** 1 Cancer Institute, Fudan University Shanghai Cancer Center, Shanghai, China; 2 Department of Oncology, Shanghai Medical College, Fudan University, Shanghai, China; 3 Department of Gynecological Oncology, Fudan University Shanghai Cancer Center, Shanghai, China; 4 Central Laboratory, the Fifth People's Hospital of Shanghai, Fudan University, Shanghai, China; National Health Research Institutes, Taiwan

## Abstract

In the present study, we investigated the *in vitro* antitumor functions of a synthetic chalcone derivative 4,3′,4′,5′- tetramethoxychalcone (TMOC) in ovarian cancer cells. We found that TMOC inhibited the proliferation and colony formation of cisplatin sensitive cell line A2780 and resistant cell line A2780/CDDP, as well as ovarian cancer cell line SKOV3 in a time- and dose-dependent manner. Treatment of A2780 cells with TMOC resulted in G_0_/G_1_ cell cycle arrest through the down-regulation of cyclin D1 and CDK4, and the up-regulation of p16, p21 and p27 proteins. We demonstrated that TMOC might induce cell apoptosis through suppressing Bcl-2 and Bcl-xL, but enhancing the expression of Bax and the cleavage of PARP-1. Treatment of TMOC also reduced the invasion and migration of A2780 cells. Finally, we found that TMOC inhibited the constitutive activation of STAT3 signaling pathway and induced the expression of the tumor suppressor PTEN regardless of the p53 status in cell lines. These data suggest that TMOC may be developed as a potential chemotherapeutic agent to effectively treat certain cancers including ovarian cancer.

## Introduction

Ovarian cancer is the leading cause of death from gynecologic malignancies in women. Due to the lack of sensitive and specific methods for early detection, nearly 60–70% of ovarian cancer patients are diagnosed at advanced stages [1], [2]. Despite advances in treatment of ovarian cancer, predominantly involving cytoreductive surgery followed by platinum-based chemotherapy, the survival rate of ovarian cancer patients remains very low [3]. Clinical problems including acquired resistance to conventional chemotherapies as well as the metastatic and invasive capabilities of the disease have severely impaired the treatment success [4], [5]. Therefore, the continued development of novel therapeutic agents for ovarian cancer, especially for the platinum resistant cells, is still urgent.

Naturally occurring products from various plants are always important in the discovery of new therapeutic agents [6], [7]. For instance, chalcone derivatives (molecules containing 1,3-diphenyl-2-3propen-1-one groups), one of the major classes of natural products with widespread distribution in spices, tea, beer, fruits and vegetables, display various interesting biological activities including anti-inflammatory, antimicrobial, antioxidant, and anticancer properties [8]–[10]. Specifically, as a structure mimics of combretastatin A-4(CA-4), 3′,4′,5′-trimethoxychalcone was reported to exhibit antimitotic properties caused by the inhibition of tubulin polymerization [11]–[14].

**Figure 1:**
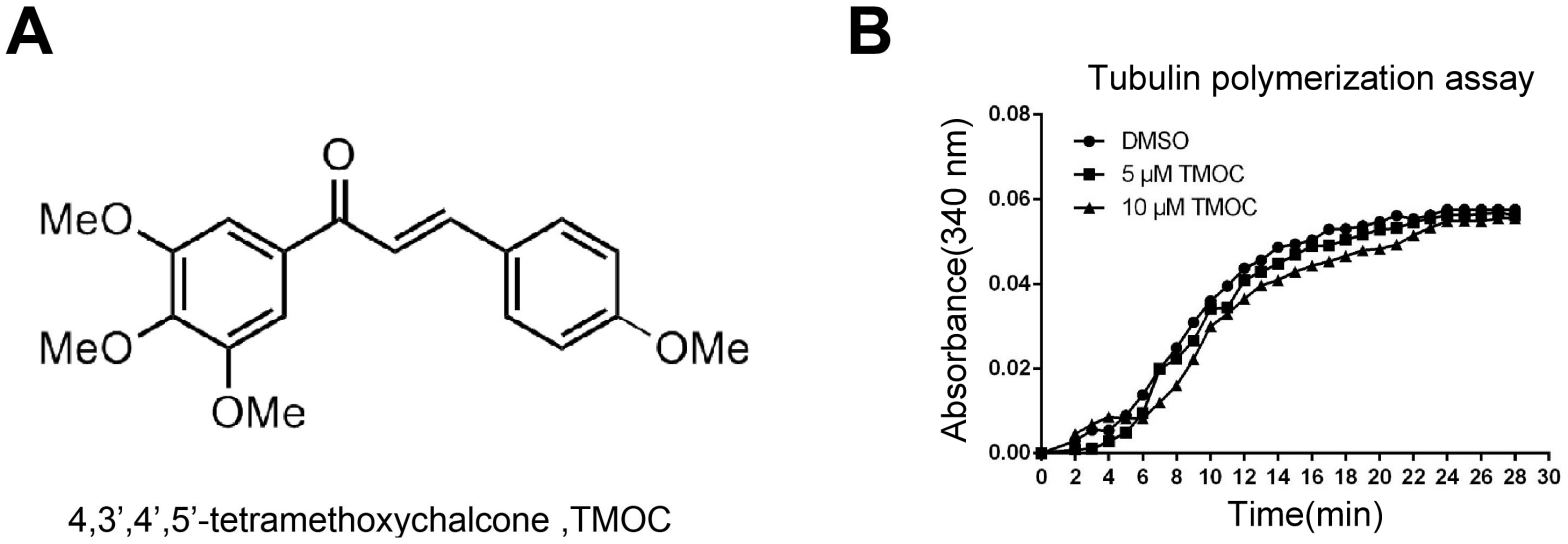
Chemical structures of 4,3′,4′,5′- tetramethoxychalcone (TMOC) (A), and TMOC did not interrupt tubulin polymerization (B).

In our efforts to discover cytotoxic agents against ovarian cancer cells, a series of CA-4 related compounds were synthesized and evaluated for their anti-proliferative activities in human epithelial ovarian cancer cell line A2780 *in vitro* (data not shown). Among these compounds, 4,3′,4′,5′-tetramethoxychalcone (TMOC, Fig. 1A) possessed the highest inhibitory potency against A2780 cells. However, subsequent *in vitro* assays showed that TMOC did not interrupt the tubulin polymerization (Fig. 1B), indicating that the anti-cancer mechanism of TMOC still remains to be elucidated.

## Materials and Methods

### Materials

TMOC was synthesized according to the previous report and was determined by spectra including ^1^H-NMR, ^13^C-NMR, and high resolution mass spectrum (HRMS) which were great agreed with the literatures [14], [15]. The purity of TMOC was more than 98% which was analyzed by HPLC.

RPMI-1640 medium and fetal bovine serum (FBS) were purchased from Thermo Scientific (South Logan, UT, USA). MTT, propidium indide (PI), 4,6-diamidino-2-phenylindole (DAPI), and the antibody to β-Actin were purchased from Sigma-Aldrich (St Louis, MO, USA). Gentian violet was purchased from Solarbio (Beijing, China). The Annexin V-FICT/PI apoptosis detection kit, invasion chambers, matrigel, and the antibody to p21 were purchased from BD Biosciences (Franklin Lakes, NY, USA). Cell lysis buffer and BCA protein assay kit were purchased from Beyotime (Shanghai, China). PVDF membrane and chemiluminescent reagents were from Millipore (Billerica, MA, USA). Antibodies to cyclin D1, CDK4, p16, p21, Bcl-xL, Bax, STAT3, p53, PTEN, c-myc were from Santa Cruz Biotechnology. Antibodies to phospho-Src (Tyr416), Src, phospho-STAT3 (Tyr705) and cleaved-PARP-1 were purchased from Cell Signaling Technology.

### Cell culture and transfection

The human epithelial ovarian cancer cell lines A2780 and SKOV3 were purchased from ATCC. The cisplatin resistant ovarian cancer cell line A2780/CDDP was kindly provided by Prof. Ling-Ya, Pan [16]. Immortalized but pre-neoplastic human ovarian epithelial cells T29 was derived from ovarian surface epithelial cell lines IOSE-29 as described previously [17]. Cells were routinely cultured with RPMI-1640 supplemented with 10% FBS, 100 U/mL penicillin and 100 µg/mL streptomycin in a humidified incubator at 37°C in an atmosphere of 5% CO_2._ For transfection studies, cells were transiently transfected with STAT3-CA (constitutive active mutant, A661C and N663C) [18], [19], STAT3-DN (dominant negative mutant, Y705F) [20] or the control vector using Fugene HD (promega). The STAT3 constructs were gifts from Dr. Hesham M. Amin (MD Anderson Cancer Center, Houston, Texas, USA).

### *In vitro* anti-proliferation assay

The *in vitro* anti-proliferative activity of TMOC was measured by the MTT reagent, as described in the literature [21]. Briefly, 5×10^3^ cells in 100 µL of medium per well were plated in 96-well plates. After incubated for 24 h, the cells were treated with different concentration of TMOC or DMSO (as negative control) for 24 h, 48 h or 72 h. Then, the medium with the compound or DMSO was replaced with 200 µL of fresh medium containing 10% MTT (5 mg/mL in PBS) in each well and incubated at 37°C for 4 h. Last, the MTT-containing medium was discarded and 150 µL of DMSO per well was added to dissolve the formazan crystals newly formed. Absorbance of each well was determined with a microplate reader (Synergy H4, Bio-Tek) at a 590 nm wavelength. The inhibition rates of proliferation were calculated with the following equation:
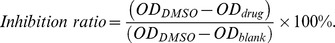



### Colony formation assay

5×10^2^ cells per well were seeded in six-well plates at a single cell density. 48 h later, the cells were treated with different concentrations of TMOC or DMSO (as negative control) for 48 h. Then the medium was replaced with fresh medium to allow cell growth for one week. The cells were fixed with methyl alcohol for 15 min and stained with gentian violet for 30 min. Colonies consisting of more than 50 cells were counted.

### Cell cycle analysis

Cell cycle status was detected by flow cytometry according to a previously published method [22], and were analyzed by Multicycle AV (for windows, version 320) software. Briefly, cells were first treated with various concentrations of TMOC or DMSO for 24 h, and then harvested, washed twice with 1× PBS, and resuspended in 200 µL of 1× PBS. The cells were fixed in 4 mL of ice-cold 75% ethanol at −20°C overnight and stained with 500 µL PI (50 µg/mL, Sigma) containing 0.1% RNase (1 mg/mL, Sigma) for 15 min in dark condition at room temperature. The cells were then analyzed by flow cytometry (Cytomics FC 500 MPL, Beckman Coulter). The results were indicated as mean values from three independent determinations.

### Cell apoptosis analysis

To detect apoptosis, cells were incubated with the different concentrations of TMOC or DMSO (as negative control) for 24 h. The cells were harvested, washed twice with cold 1×PBS, and resuspended in 200 µL binding buffer at the density of 1×10^5^ cells/mL. The cells were then stained with 5 µL Annexin-V and PI, for 15 min in dark condition at room temperature and subjected to analysis by flow cytometry. The early apoptosis was evaluated based on the percentage of cells with Annexin V+/PI−, while the late apoptosis was that of cells with Annexin V+/PI+. The results were indicated as mean values from three independent determinations.

### DAPI and PI staining

Nuclear morphological and membrane integral changes of apoptosis were determined by DAPI and PI staining respectively, as described previously [23], [24]. 3×10^5^ cells were seeded in 6-well plates and cultured for 48 h, followed by treatment with diluent or with desired concentrations of TMOC for 24 h. For DAPI staining, cells were washed with PBS for three times, fixed with menthol, and permeabilized with 0.1% Triton X-100, followed by staining with DAPI (1∶2000 dilution, in 1x PBS) at 37°C for 15 min in dark. For PI staining, cells were washed by PBS three times and directly stained with PI at 37°C for 15 min in the dark. After staining, the cells were washed with PBS to remove unbound dye (PI) and observed using fluorescent microscopy (Olympus). Fluorescent images were recorded using a cooled CCD camera.

### Wound healing assay

To detect cell motility, cells were seeded in 6-well plates and grown to 90% confluence. A single scratch wound was created on monolayer cells by using a sterile micropipette tip. Subsequently, medium with cellular debris was removed, and fresh serum-free medium (without FBS supplementation) containing different concentrations of TMOC or DMSO was added. Cells were incubated at 37°C and pictures of each wounded monolayer were taken at 0, 36 and 72 h.

### Transwell invasion assay

Cell invasion was assayed using chambers pre-coated with matrigel [25]. Briefly, 5×10^4^ cells in 300 µL serum-free RPM-1640 were seeded into the upper chamber. The chamber was placed into a 24-well plate and the lower wells contained RPM-1640 medium with 10% FBS and different concentrations of TMOC or DMSO (as a negative control). After 24 h of incubation, the cells on the upper surface of chamber were carefully swabbed with a cotton swab. The cells migrated through the chamber were fixed with methanol, stained with crystal violet and subsequently counted from 5 different areas from each well under inverted microscope. At least three independent experiments were performed.

### Western blot analysis

Cells were treated with different concentrations of TMOC or DMSO (as a negative control) for 24 h, and then were harvested, washed with cold 1×PBS twice, lysed with cell lysis buffer for 30 min on ice, and centrifuged at 12,000 rpm for 15 min at 4°C. The concentration of total protein was determined by BCA protein assay kit. Equal amounts (30 µg per load) of protein samples were subjected to SDS-PAGE electrophoresis and transferred on to polyvinylidene fluoride (PVDF) membranes which was then blocked in 10% non-fat milk, and reacted with primary antibodies. After incubation with the secondary antibodies conjugated with horseradish peroxidase (HRP), the protein bands were developed with the chemiluminescent reagents.

### Statistical analysis

The data were calculated using Graph Pad Prism and expressed as mean ± S.E. The values of IC_50_ were fitted using a nonlinear regression model with a sigmoidal dose response. Comparisons between control and treated groups were determined by paired *t* test or one-way ANOVA followed by Tukey's multiple comparison tests. Results were considered statistically significant at the *p*<0.05 level.

## Results

### TMOC suppresses cell growth

**Figure 2:**
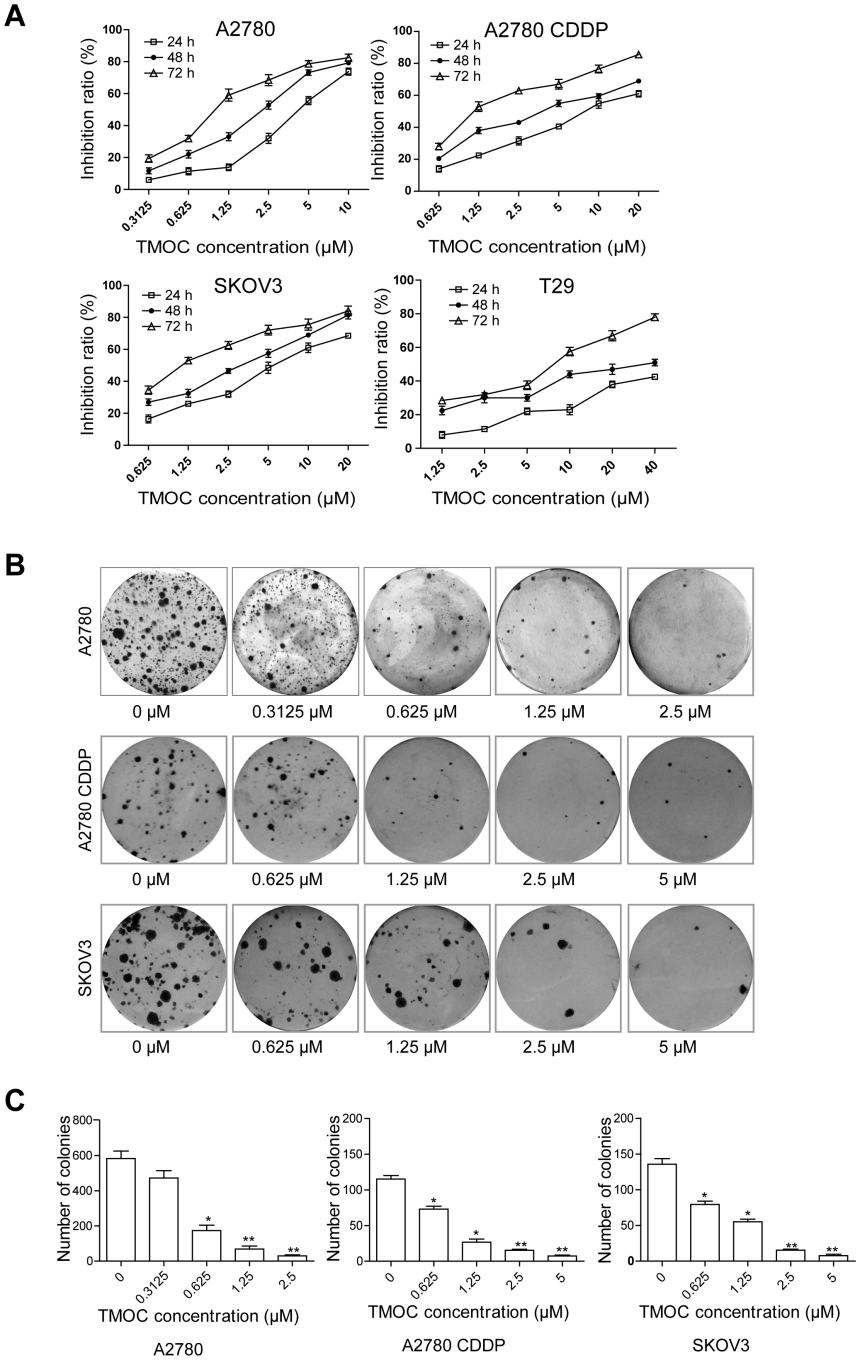
The effect of TMOC on cell proliferation and colony formation. (A) TMOC inhibited cell proliferation in dose- and time-dependent manner. Cell viability was determined by MTT assays. (B) Representative images of cell colonies after treatment with various concentrations of TMOC for 24 h. (C) Colony formation rate after treatment with TMOC for 24 h.

We first determined the anti-proliferative effects of TMOC on human ovarian carcinoma cells, including A2780 (p53 wild-type), A2780/CDDP (cisplatin resistant subline of A2780, p53 mutant) and SKOV3 (p53 null) cells, as well as pre-neoplastic ovarian epithelial T29 cells. Cells were treated with various concentrations (ranging from 0.3125 to 40 µM) of TMOC for 24, 48, and 72 hours. As shown in Fig. 2A, treatment of A2780 cells with TMOC resulted in a corresponding decrease of cell proliferation and viability in a dose- and time-dependent manner. Similar effects were obtained on treatment of A2780/CDDP and SKOV3 cells (Fig 2A). In contrast, the sensitivity of T29 cells to TMOC was much low, as the concentration of TMOC effective on T29 cells was detected at 40 µM in 72 h. The IC_50_ values were calculated and listed in Table 1. Thus, these data suggest that TMOC does have a cytotoxic effect on ovarian tumor cells regardless p53 status, but processes less cytotoxicity in pre-neoplastic ovarian epithelial cells. Moreover, we also tested the anti-proliferative activities of chalcone (1,3-Diphenyl-2-propen-1-one), which is the mother compound of TMOC (Table 1). Compared with TMOC, chalcone exhibited much weak effect on the inhibition of both neoplastic and pre-neoplastic cell growth, and presented poor water solubility, which may indicate that the four methyloxy groups are essential for the anti-cancer activities and physiochemical properties.

**Table 1:** The IC_50_ values and water solubility of TMOC and chalcone.

			TMOC	chalcone
IC_50_ (µM.)	A2780	24 h	3.5	>100
		48 h	1.3	>100
		72 h	0.6	95.4
	A2780/CDDP	24 h	8.5	> 100
		48 h	4.3	>100
		72 h	0.9	>100
	SKOV3	24 h	5.2	> 100
		48 h	3.6	>100
		72 h	1.1	>100
	T29	24 h	>40	>100
		48 h	30.1	>100
		72 h	7.4	>100
Water solubility (µg/mL)			4	<1

In the following experiments, we determined the effect of TMOC on the colony formation ability of the ovarian cancer cell lines. Colony formation assay is an *in vitro* cell survival assay based on the ability of a single cell to proliferate indefinitely, thereby retaining its reproductive ability to form a colony consisting of at least 50 cells. Colony formation assay is the method of choice to determine cell reproductive death after treatment with cytotoxic agents, and now widely used to determine the cytotoxicity induced by various chemotherapeutic agents [26]. In this study, as shown in Fig. 2B, treatment of A2780, A2780/CDDP, and SKOV3 cells with TMOC at concentrations of 0.3125, 0.625, 1.25 and 2.5 µM for 48 hours dose-dependently inhibited colony formation, when compared to treatment with diluent (DMSO). The numbers of colonies formed by cells treated with TMOC or diluent were summarized in Fig 2C, which confirmed the inhibited effect of TMOC on the growth of ovarian cancer cells.

### TMOC induces G_0_/G_1_ phase cell cycle arrest

**Figure 3:**
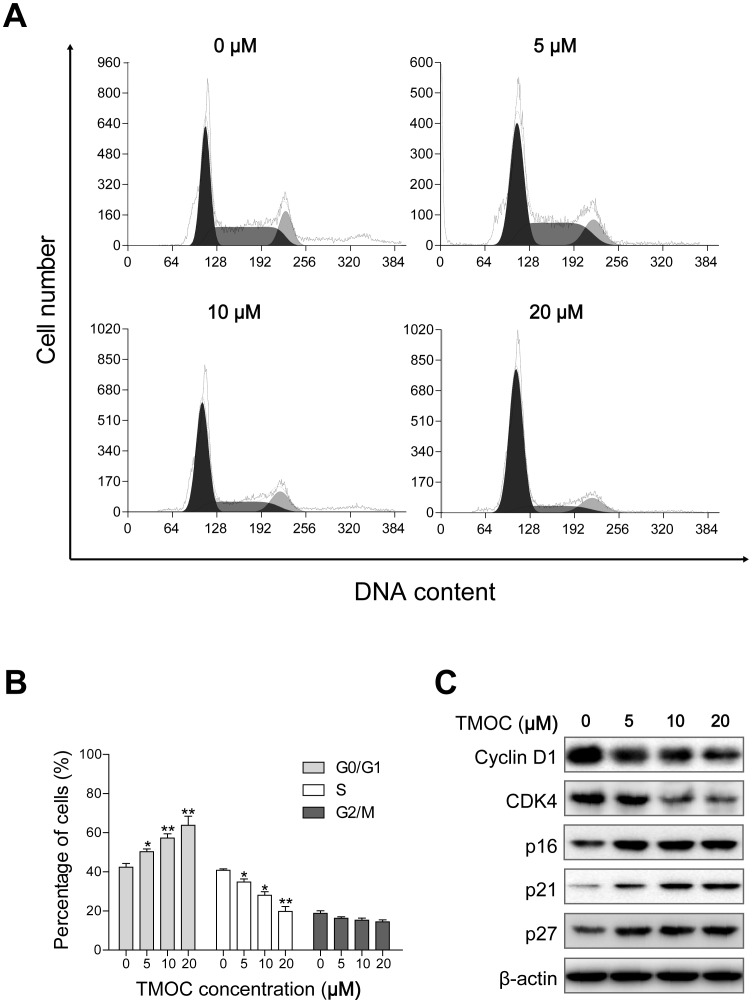
The effect of TOMC on cell cycle progression. (A) Cell cycle distribution after treatment with different concentrations of TMOC for 24 h. (B) Quantitative analysis of TMOC-treated cells. (C) Regulation of cell cycle associated proteins in A2780 cells. The experiments were repeated three times, and a representative experiment is shown. * *p*<0.05, ** *p*<0.01 compared to control.

Chemical anti-tumor agents can inhibit cell proliferation through induction of cell cycle arrest. Therefore, to understand how the cell growth was inhibited by TMOC, the cell cycle progression was analyzed by quantitating DNA content using flow cytometry. We treated A2780 cells with TMOC for 24 h and examined the DNA content by propidium iodide (PI) staining (Fig. 3A). As illustrated in Fig. 3B, compared with control cells treated with the diluent, when A2780 cells were treated with high concentrations of TMOC at 5, 10 and 20 µM, the percentage of cells in G_0_/G_1_ phase was elevated from 42.3% to 64.1%, and the percentage of cells in S and G_2_/M phase was decreased concomitantly. Because cell cycle progression is regulated by cyclin/cyclin-dependent kinase (CDK) complexes, the uncontrolled expressions of cyclins and/or CDKs may lead to cell cycle dysregulation and tumorigenesis [27]. Therefore, we examined whether TMOC affected cyclins or CDKs expressions. As shown in Fig. 3C, treatment of cells with TMOC down-regulated the expressions of cyclin D1 and CDK4, but up-regulated the expressions of p16, p21^Cip1^ and p27^Kip^ in a dose-dependent manner. Taken together, the altered expressions of the cell cyclins and CDK inhibitors might be associated with cell cycle arrest caused by TMOC.

### TMOC induces cellular apoptosis

**Figure 4:**
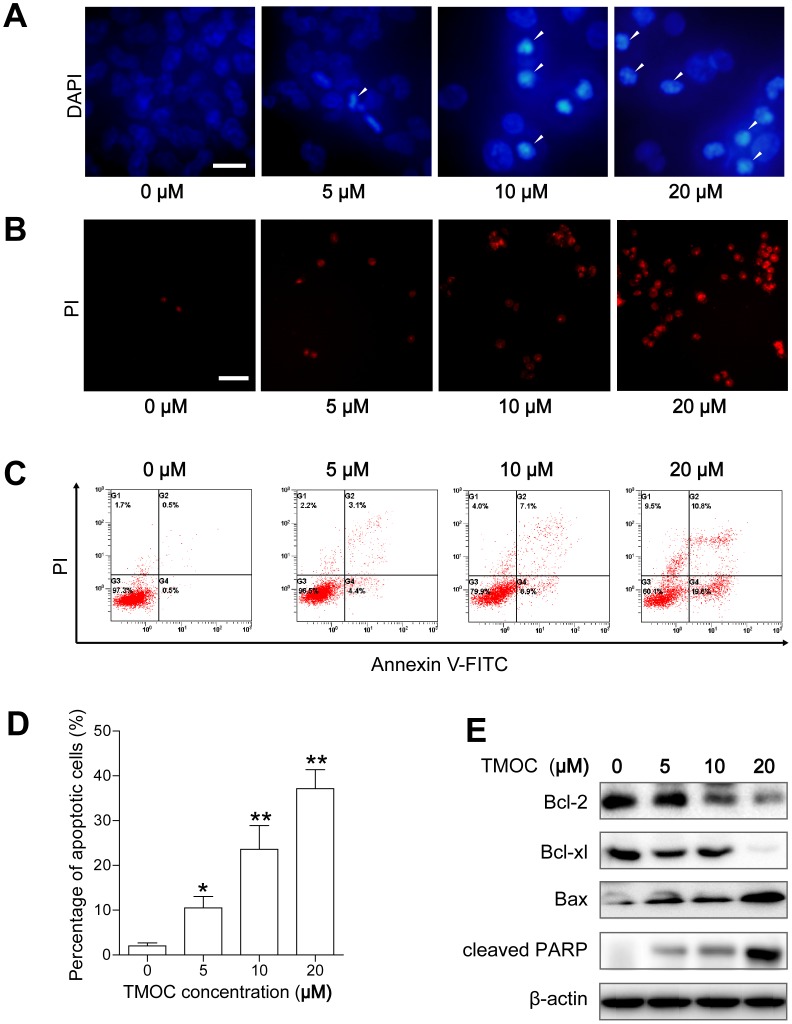
Apoptosis induced by TMOC. (A) DAPI nuclei staining was used to visualize the apoptosis induced by TMOC. Arrows represent the apoptotic cells. Scale bar = 10 µm. (B) PI uptake was analyzed under a fluorescent microscope. Scale bar = 50 µm. (C) Representative flow cytometry profiles of apoptosis and (D) quantitative results obtained using Annexin V/PI staining. (E) Western blots of apoptotic related proteins. The assay was repeated three times, and a representative result is shown. * *p*<0.05, ** *p*<0.01 compared to control.

DAPI nuclei staining was used to visualize the apoptosis induced by TMOC. As shown in Fig. 4A, the nuclear apoptosis of TMOC-treated A2780 cells were characterized by condensed and fragmented chromatin stained with strong blue fluorescent dots, whereas control cells were stained with uniform blue [24]. In addition, the apoptosis was also indicated by staining with PI, a membrane impairment nuclear dye, as shown in Fig. 4B. These results indicated that TMOC could induce cellular apoptosis. To further determine the number and stage of apoptotic cells, Annexin-V/PI double staining was applied to quantify the number of apoptotic cells treated with TMOC [22]. As illustrated in Fig. 4C and 4D, the total proportions of cells stained with Annexin V+/PI^−^ (the right lower quadrant representing early apoptosis) and Annexin V^+^/PI^+^ (the right upper quadrant representing late apoptosis and necrosis) cells were increased from 1.2% to 35.5% after treatment of A2780 cells with TMOC at 5, 10 and 20 µM for 24 h. These data further confirmed that TMOC could strongly induce cellular apoptosis of ovarian cancer cells in a dose-dependent manner.

Next, we investigated the signaling pathway involved in TMOC induced apoptosis by Western blot analysis. We showed that (Fig. 4E), TMOC up-regulated the expression of the pro-apoptotic protein Bax, but down-regulated the expressions of the anti-apoptotic proteins Bcl-2 and Bcl-xL in a does dependent manner. Moreover, compared with control cells, exposure to TMOC induced the cleavage of poly(ADP-ribose) polymerase-1 (PARP-1), a marker of cells undergoing apoptosis [28]. These results suggested that TMOC might induce cellular apoptosis through these proteins.

### TMOC suppresses cell migration and invasion

**Figure 5:**
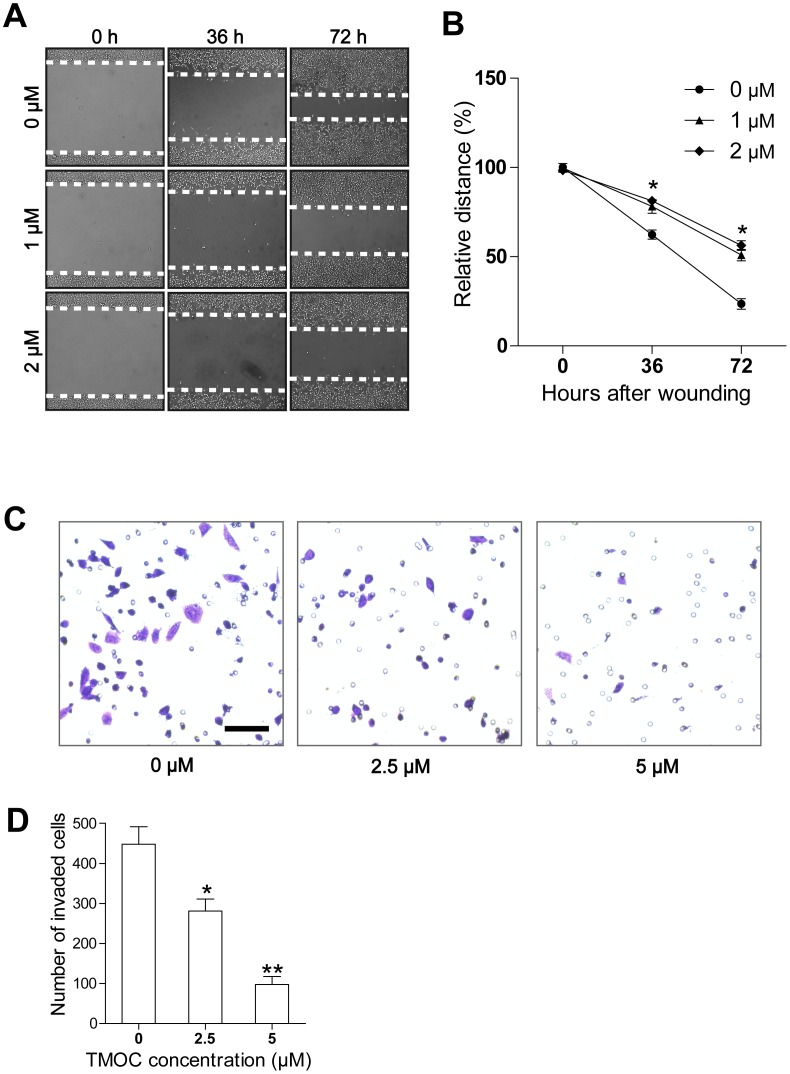
The inhibitory effect of TMOC on cell migration and invasion. (A) TMOC inhibited cell migration. Representative photographs of the cells were taken at time 0, 36 or 72 h. (B) Quantification of wound healing. The experiments were repeated three times. (C) Representative photographs of invasive cells in transwell invasive assays. Scale bar = 20 µm. (C) Quantification of invasive A2780 cells. The experiments were repeated three times. * *p*<0.05, ** *p*<0.01 compared to control.

Metastatic spreading, one of the features in ovarian cancer cells, is an important factor to low the survival rate of patients [29], while anti-cancer agents can not only inhibit cancer cell growth, but may also halt cell metastases. In this study, to determine whether TMOC suppresses invasion and migration in ovarian cancer cells, we performed wound healing and transwell invasion assays. In wound healing assay, after a single wound was scratched on monolayer of A2780 cells, the medium was changed with serum-free medium containing TMOC or diluent (as control) in order to avoid the influence of cell proliferation. As shown in Fig. 5A and 5B, the migration of cells was significantly decreased by TMOC. The suppression of migration was not due to apoptosis or growth arrest as the concentration of TMOC at 0.5 µM or 1 µM only induced a relative low toxicity in A2780 cells. The results obtained from the transwell assay, showed that TMOC inhibited the invasion and migration of A2780 cells in a dose-dependent manner (Fig. 5C and 5D).

### Signaling pathways involved in TMOC-mediated anti-cancer effects

**Figure 6:**
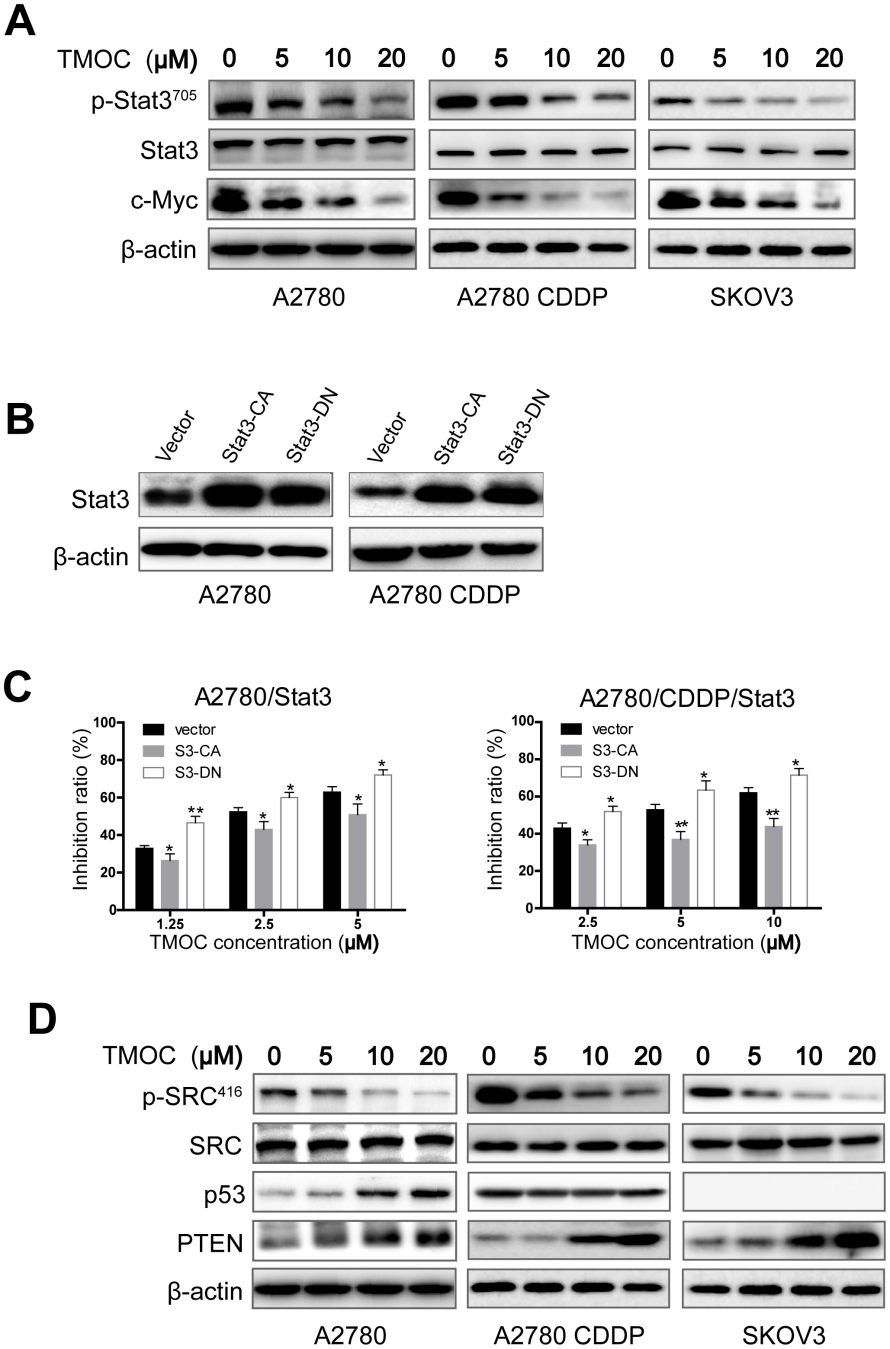
The molecular mechanism of *in vitro* anti-tumor effects of TMOC. (A) TMOC treatment inhibited the STAT3 phosphorylation and down-regulated the level of c-myc in a dose dependent manner. (B) A2780 and A2780/CDDP cells were transfected with STAT3 constitutively active (S3-CA) plasmid, STAT3 dominant negative (S3 DN) plasmid or control vector. (C) Introduction of S3-CA significant blocked the anti-proliferative activity of TMOC. In contrast, introduction of S3-DN sensitized the cancer cells to TMOC treatment. (D) TMOC inhibited the c-Src kinase phosphorylation and up-regulated the tumor surpressor PTEN in all of the three cell lines, but only up-regulated p53 in A2780 cells. β-actin was used as an equal loading control. The experiments were repeated three times and a representative experiment is shown. * *p*<0.05, ** *p*<0.01 compared to control.

Signal transducer and activator of transcription-3 (STAT3), an oncogenic transcription factor, is often constitutively active in human cancer cells [18]. Recent studies have shown that the activation of STAT3 plays a pivotal role in the survival, hyper-proliferation, and metastatic progression of ovarian cancer [30], [31]. Once activated, the phosphorylated STAT3 may up-regulate the expression of genes such as apoptosis inhibitors (Bcl-xl, Bcl-2), cell cycle regulators (cyclin D1) and oncogenic transcription factors (c-myc) in tumorigenesis [32], [33]. Therefore, we employed western blot to test whether the suppression of STAT3 signaling pathway was involved in the TMOC-mediated anti-cancer effects. Indeed, as shown in Fig. 6A, with the increase of TMOC concentration, the phosphorylation of STAT3 as well as the expression of c-myc, a known downstream target of STAT3 [33], [34], was dose-dependently suppressed in A2780, A2780/CDDP and SKOV3 cells, whereas no effect on the expression levels of total STAT3 was observed.

To further confirm the role of STAT3 in TMOC-induced cytotoxicity, we transfected A2780 and A2780/CDDP cells with STAT3 constitutively active (S3-CA) plasmid, STAT3 dominant negative (S3-DN) plasmid or control vector, respectively, and then determined the anti-proliferative effect of TMOC on the transfected cells. As shown in Fig. 6C, introduction of S3-CA significantly resecued the anti-proliferative effect of TMOC. In contrast, introduction of S3-DN sensitized cancer cells to TMOC treatment. Collectively, these data suggest that TMOC may elicit its anti-cancer activity through inhibition of STAT3 signaling pathway.

Several studies have demonstrated that the constitutive activation of STAT3 is often trigged by the non-receptor tyrosine kinase c-Src and negatively regulated by the tumor suppressors p53 and PTEN [35], [36]. Thereby, we also examined whether TMOC could suppress the activation of c-Src kinase and modulate the expression of p53 and PTEN in the three ovarian cancer cell lines. As shown in Fig. 6D, we found that TMOC dose-dependently inhibited the phosphorylation of c-src kinase, whereas the total level of c-src remained unchanged. Meanwhile, TMOC up-regulated the expression levels of PTEN in all of the three cell lines, but only up-regulated p53 in A2780 cells.

## Discussion

In an attempt to develop novel chemotherapy agents with less harmful side-effects to treat cancer, natural products and their synthetic analogs have received high attention [37]. For instance, many studies have shown that several chalcone compounds, both derived from nature and synthetic versions, exhibit cytotoxic and antitumor activities [38]. In this study, we intended to study the anti-cancer activity and the underlying mechanism of TMOC, a synthetic chalcone compound, in ovarian cancer cell lines. We showed that TMOC significantly inhibited the proliferation and colony formation of A2780, A2780/CDDP, and SKOV3 cells (Fig. 2), which suggests that TMOC may be an effective chemotherapeutic agent against both cisplatin sensitive and resistant ovarian cancer cells. Importantly, we found that TMOC exhibited less toxicity to pre-neoplastic human ovarian epithelial cells than to neoplastic cells under the same concentration.

We found that TMOC induced G_0_/G_1_ cell cycle arrest through the down-regulation of cyclin D1 and CDK4, and the up-regulation of p16, p21 and p27 proteins (Fig. 3C). The cyclin D1/CDK4 complex is responsible for cell cycle progression in early G_1_ phase and is frequently overexpressed in various human carcinomas including ovarian cancer [39]–[41]. The p16 protein is a specific inhibitor of CDK-cyclin D complex, preventing the phosphorylation of Rb and cell cycle reentry at G_0_/G_1_ phase [39]. p21 and p27, which belong to Cip/Kip family, negatively regulate the cell cycle progression through inhibition of CDK-cyclin complexes [42].

Apoptosis is normally a balanced system tightly regulated by anti-apoptotic and pro-apoptotic effectors, including proteins of the Bcl-2 family. The anti-apoptotic proteins Bcl-2 and Bcl-xL promote cell survival whereas the pro-apoptotic protein Bax induces the programmed cell death. The ratio of Bax/Bcl-2 is critical for the induction of apoptosis and determines whether cells will undergo apoptosis [43]. In the present study, TMOC treatment resulted in the increase of Bax but led to the decrease of Bcl-2 and Bcl-xL(Fig. 4E). The increase of the Bax/Bcl-2 ratio from 0.07 to 1.90 may be responsible for the concomitant apoptosis due to the disruption of mitochondrial membrane potential and the inactivation of key cellular proteins such as PARP-1.

Accumulating studies provide strong evidence that the STAT3 activation has been linked with a variety of tumors including multiple myeloma, ovarian cancer, breast cancer, prostate cancer, and so on [44]. Thus, the suppression of STAT3 signaling pathway has emerged as an effective way for cancer therapy. In our present investigation, the results from Western blots showed that the phosphorylation of STAT3 and its upstream protein tyrosine kinases c-Src (Fig. 6A and 6D) was inhibited by TMOC, which was also supported by the down-regulation of the transcriptionally regulated targets such as cyclin D1, Bcl-xL and c-myc [33]. Besides, we identified that TMOC could up-regulate the expression of PTEN in all of the three cell lines but only up-regulated the expression of p53 in A2780 cells. Although, p53 is reported to negatively regulate the phosphorylation and DNA binding activity of STAT3 through facilitating the tumor suppressor PTEN [36], [45], our data revealed that TMOC elicited anti-proliferation function and inhibited the phosphorylation of STAT3 regardless p53 status, which may indicate that the anti-cancer activity of TMOC is p53-independent.

In conclusion, we provide strong evidence that TMOC inhibits cell growth and motility, and induces cell cycle arrest and apoptosis of human ovarian cancer cells through repressing STAT3 and c-Src activation. However, further study may be needed to validate the potential application of TMOC in cancer treatment.
